# The Development of Smoked Mackerel with Reduced Sodium Content

**DOI:** 10.3390/foods11030349

**Published:** 2022-01-26

**Authors:** Iga Rybicka, Marlene Silva, Amparo Gonçalves, Helena Oliveira, António Marques, Maria José Fernandes, Maria Helena Fernandes, Cristina Mateus Alfaia, Maria João Fraqueza, Maria Leonor Nunes

**Affiliations:** 1 Interdisciplinary Centre of Marine and Environmental Research, University of Porto, Terminal de Cruzeiros do Porto de Leixões, Av. General Norton de Matos S/N, 4450-208 Matosinhos, Portugal; amparo@ipma.pt (A.G.); helaoliveira@gmail.com (H.O.); amarques@ipma.pt (A.M.); nunes.leonor@gmail.com (M.L.N.); 2 Institute of Quality Science, Poznań University of Economics and Business, al. Niepodległości 10, 61-875 Poznań, Poland; 3 Portuguese Institute for the Sea and Atmosphere, Division of Aquaculture, Upgrading and Bioprospecting, Av. Alfredo Magalhães Ramalho 6, 1495-165 Lisboa, Portugal; marlene.lisboa97@gmail.com; 4 Instituto Superior Técnico, University of Lisbon, Av. Rovisco Pais 1, 1049-001 Lisboa, Portugal; 5 CIISA-Centre for Interdisciplinary Research in Animal Health, Faculty of Veterinary Medicine, University of Lisbon, Avenida da Universidade Técnica, 1300-477 Lisboa, Portugal; adanaritah@fmv.ulisboa.pt (M.J.F.); helenafernandes@fmv.ulisboa.pt (M.H.F.); cpmateus@fmv.ulisboa.pt (C.M.A.); mjoaofraqueza@fmv.ulisboa.pt (M.J.F.)

**Keywords:** mackerel, smoking, potassium chloride (KCl), salt substitute, sodium (Na), sodium chloride (NaCl)

## Abstract

The World Health Organization recommends reducing salt (sodium chloride, NaCl) intake by 30% by 2025. Since smoked fish can deliver up to 4 g NaCl/100 g, the aim of this study was to develop safe, healthy and attractive smoked chub mackerel (*Scomber japonicus)* with a reduced NaCl content. Two brines (5% and 10%) were used with different ratios of NaCl and potassium chloride (KCl). In each brine, 0%, 25%, 50% and 75% of NaCl was replaced by KCl, resulting in 1.3, 1.1, 0.9 and 0.6 g NaCl (5% brine), and 2.6, 2.0, 1.2 and 0.8 g NaCl (10% brine) per 100 g, respectively. Similar yield, nutritional, safety, texture and colour properties were found in most formulations. The most desirable taste attributes (negligible bitterness and adequate saltiness) were obtained with a 5% brine prepared with 75% NaCl + 25% KCl. Such conditions seemed to allow for obtaining an attractive product for *conscious consumers*.

## 1. Introduction

The market of processed fish and shellfish is growing and is expected to continue to grow at a 4.4% compound annual growth rate (CAGR) through 2021–2025. Every year, 600–800 million tonnes of mackerel are consumed in the European Union [1]. In 2018, half of the consumption was reported for Germany (50,000 tonnes), the United Kingdom (35,000 tonnes) and France (21,000 tonnes). Another third of global consumption belonged to (in descending order) Poland, the Netherlands, Italy, Spain, Romania, Belgium, the Czech Republic, Portugal and Hungary [2]. Mackerel’s strong position in the seafood market results from its accessibility (availability throughout the year) and affordability (low-to-moderate price). The most popular species are Atlantic mackerel (*Scomber scombrus*) and Chub mackerel, also known as Pacific mackerel (*Scomber japonicus).* Non-smoked fresh mackerel is regarded as a good source of energy (180–210 kcal/100 g), easily digestible protein (18–24 g/100 g) and fat (11–14 g/100 g) [3,4]. It also delivers a significant amount of omega-3 fatty acids (mainly docosahexaenoic acid), vitamins (e.g., vitamin D and B_12_) and minerals (e.g., selenium and zinc) [5]. The greatest nutritional benefits are attributed to two species from Alaska (Atlantic mackerel and Atka mackerel). On the other hand, *king mackerel* (*Scomberomorus cavalla*), which can be found in the western Atlantic Ocean and the Gulf of Mexico, should be avoided due to its usual high content of mercury [6].

Mackerel is in the top five *in the assortment of smoked fish* in numerous countries worldwide, such as Spain, the United States and Norway [7]. The nutritional value of smoked mackerel differs from fresh mackerel, particularly in a more dense proximate composition due to water loss caused by salting and smoking processes [8]. Moreover, smoked products, including mackerel, have a high content of salt (sodium chloride, NaCl). Salting, mostly with NaCl, acts not only as a preservative method, which extends the product’s shelf-life, but, more importantly, it also assures the sensory attributes, such as flavour and texture. NaCl is a major carrier of sodium (Na) (one gram of NaCl corresponds to 254 mg of Na), where excessive intake increases blood pressure and can lead to cardiovascular diseases, such as heart disease and stroke [9]. In general, raw fish and shellfish are not an important source of Na, and most species do not contain more than 200 mg of Na in 100 g. However, after processing, which usually includes salting, its content can be ten times higher than in fresh fish [10].

Salt reduction in food is one of the highest priorities to maintain worldwide population health. Current recommendations of the WHO suggest a maximum consumption of 5 g (a spoon) of NaCl per day, while the actual intake is 8–12 g in most European countries [11]. It is estimated that lowering Na intake could save around 40 million lives over 30 years [9]. The WHO intends to reduce salt intake by 25% until 2030 (in comparison to 2010 salt levels). Different strategies for salt reduction management include monitoring the population salt intake, governmental policies, co-operation with the food industry, social campaigns and consumer education. One of the most popular strategies is the reformulation of products at the industrial level. Despite the initiatives in the seafood industry being less developed than in the assortments of bakery or meat products, several directions have also become visible for this industry. Several studies were performed to decrease the NaCl contents by using different substitutes, such as KCl, MgCl_2_, CaCl_2_ and K-lactate [12]. KCl is used most often and effectively due to similar functionalities to NaCl. However, only partial replacement of NaCl by KCl is advisable, mainly due to its weaker salty taste and higher bitterness compared with NaCl [13]. Several experimental studies using KCl in smoked fish can be found in the literature, but so far, no research has focused on smoked mackerel with reduced Na content [14,15]. Therefore, this study was aimed at the development and quality assessment of smoked chub mackerel with reduced NaCl/Na content.

## 2. Materials and Methods

### 2.1. Raw Material

Chub mackerel was caught by commercial fisheries in the Atlantic Ocean, near Peniche, Portugal, in December 2020. Fish were kept on ice at 4 °C during transportation and were shipped to the laboratory within 12 h. All fish were coded and biometric data was collected (n = 27; mean weight: 392 ± 61 g), then the fish was gutted, washed with tap water, filleted, washed and drained. Both fillets from each fish were individually packed in plastic bags (coded with the respective fish code) and frozen at −20 °C. Later, all fillets (n = 54; mean weight: 153 ± 19 g) were thawed at 4 °C for 24 h, and six random fillets (from three different fish) were analysed as raw material. The remaining fillets (n = 48 corresponding to 24 fish) were subjected to further salting and smoking processing.

### 2.2. Salting and Smoking

**Figure 1:**
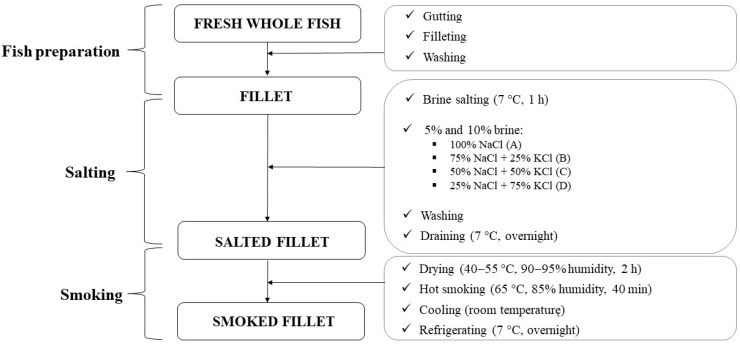
Scheme of the development of the smoked mackerel formulations.

The development of the smoked mackerel is summarised in Figure 1.

**Figure 2:**
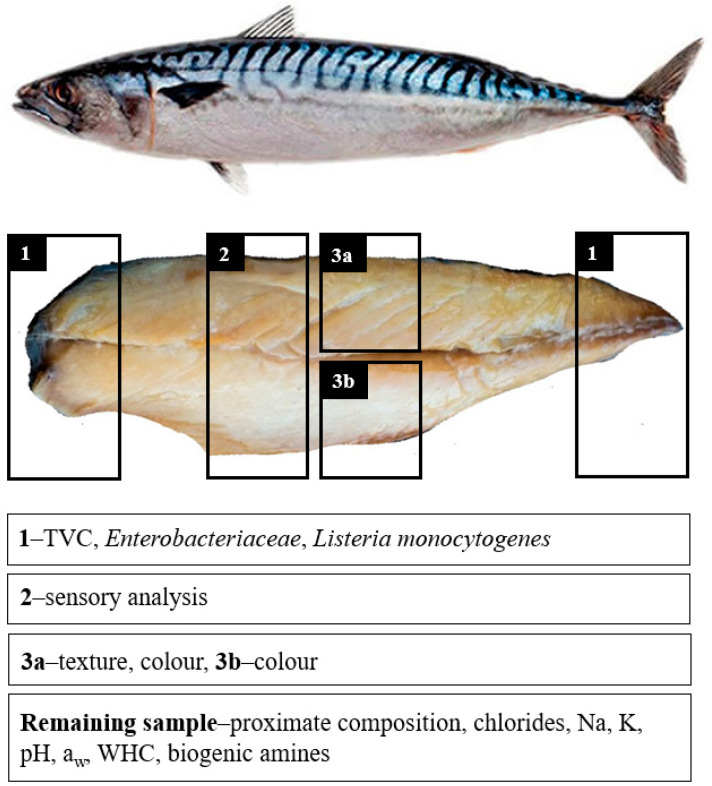
Smoked mackerel sampling for analysis.

Thawed fillets were immersed in brines of different concentrations and combinations of NaCl and KCl. The conditions (brine strength and time) were chosen in preliminary experiments based on [16]. In total, eight formulations were prepared: half of the samples used a 5% brine and the second half used a 10% brine (n = 6 fillets (3 × 2 fillets from the same fish) were used for each formulation). For each brine, four formulations were prepared: 100% NaCl (formulation A), 75% NaCl + 25% KCl (B), 50% NaCl + 50% KCl (C) and 25% NaCl + 75% KCl (D), where the NaCl and KCl levels were calculated based on the molecular weight; six fillets were assigned to each formulation. Food grade NaCl (Enisal, Barcelona, Spain) and KCl (Quimics Dalmau S.L., Barcelona, Spain) were used. The fillets were placed in bulk in the brine where they remained without stirring for 1 h at 7 °C. Then, the fillets were withdrawn from the brine, quickly washed with distilled water, left to drain overnight at 4 °C (following the methodology described in [17]) and hot smoked in a smoking chamber (Simia, Simia-Soc. Industrial de Máquinas para a Indústria Alimentar, Lda., Montijo, Portugal). The following steps were applied: drying at 40–55 °C with 90–95% humidity over 2 h and hot smoking at 65 °C with humidity of 85% for 40 min. After smoking, the fillets were left to cool to room temperature and refrigerated at 7 °C overnight. The collection of samples (in both fillets of each fish) for sensory, microbiological, colour and texture analyses was performed on the same day and as shown in Figure 2. Between the analyses, the samples were kept in plastic bags in the refrigerator (7 °C). The remaining parts of the fillets (without skin and bones) were combined into one sample, homogenised for 1 min using a blender (Moulinex, Écully, France), packed in plastic bags and frozen at −20 °C. The samples were stored for up to four weeks until the physical–chemical analyses. Before the analyses, the samples were thawed at 4 °C for 24 h.

### 2.3. Yield

The yield was calculated from the weight of each fillet according to the following formula:

(1) $$\mathrm{Yield}\;\left[\%\right]=\frac{\mathrm{weight}\;\mathrm{before}\;\mathrm{processing}}{\mathrm{weight}\;\mathrm{after}\;\mathrm{processing}}\times 100$$

where the before processing applied to the weight before salting and after processing to the weight after smoking.

### 2.4. Proximate Composition (Moisture, Fat, Protein)

Moisture and fat contents were determined according to the Association of Official Analytical Chemists methods [18]. Moisture was determined by drying at 105 ± 1 °C in the oven until a constant weight (ULE 500, Memmert, Schwabach, Germany). Free fat was determined through the Soxhlet extraction method in a Soxhlet apparatus (Behr Labor-Technik, Dusseldorf, Germany) using diethyl ether solvent (at approximately 40 °C; 7 h) and by weighing the fat residue after drying (105 ± 1 °C) in an oven. Crude protein was calculated from total nitrogen using the conversion factor of 6.25 [19]. Total nitrogen was analysed according to the Dumas method [20] in an automatic nitrogen analyser (LECO FP-528, LECO Corp., St. Joseph, MO, USA) calibrated with EDTA. Nitrogen was released via combustion at 850 °C and detected using thermal conductivity. All analyses were performed in triplicate.

### 2.5. Sodium (Na), Potassium (K), Sodium Chloride (NaCl) and Chloride (Cl^−^)

The contents of Na and K were analysed using microwave plasma-atomic emission spectrometry (MP-AES 4210, Agilent Technologies, Melbourne, Australia) after prior mineralisation in a microwave oven (CEM 6, Mars, CEM Corporation, Matthews, NC, USA). Briefly, 0.5 g of the sample was mixed with HNO_3_ (65%) and H_2_O_2_ (30%) [21]. After mineralisation, the solutions were filled to 50 mL with demineralised water (Hydrolab System, Wiślina, Poland). Three digestions were performed for each sample. The spectroscopic determinations were performed at analytical wavelengths of 330.3 nm for Na and 404.4 nm for K [22]. The NaCl content was calculated through the Na levels according to the following formula:

(2) $$X=\frac{K\times Y}{1000\times Z}$$

where *X* is the salt content (g/100 g), *K* is the NaCl molar mass (58.44 g/mol), *Y* is the Na level (mg/100 g) and *Z* is the Na molar mass (22.99 g/mol). The Cl^−^ content was determined in triplicate using a volumetric titration of ~2 g of sample with silver nitrate (AgNO_3_) according to Mohr’s method [23].

### 2.6. pH, Water Activity (a_w_) and Water Holding Capacity (WHC)

The pH values were measured using a pH meter (Hanna FC200, Hanna Instruments, Inc., Woonsocket, RI, USA) by inserting the electrode for solids directly into the fillet. The a_w_ was determined at 20 °C using a water activity meter (Rotronic-Hydrolab, Rotronic Measurement Solutions, Bassersdorf, Schweiz). WHC was determined as described in [24]. Briefly, a sample of 2 g and two weighed Whatman filter papers were placed in a tube and centrifuged at 3000× *g* for 10 min at 18 °C (Kubota 6800, Kubota Corp., Tokyo, Japan). After centrifugation, the sample was removed and the filter papers were weighed again. WHC was expressed as grams of water retained per 100 g of water initially present in a sample. All analyses were performed in triplicate.

### 2.7. Total Viable Counts (TVC), Enterobacteriaceae and Listeria Monocytogenes

Samples were aseptically taken from each fillet (Figure 2) and weighed until obtaining a 10 g portion, which was prepared according to the guidelines of the ISO 6887-1:2017. The TVC analysis was performed according to ISO 4833-1:2013 (total mesophilic flora) via plating in Tryptone Glucose Extract Agar (Sharlab, Spain), followed by incubation for 48 h at 30 °C. Enterobacteriaceae were determined on Violet Red Bile Glucose Agar (VRBGA) (Sharlab, Spain) incubated at 37 °C for 24 h in microaerophilia (ISO 21528-2:2017). The *Listeria monocytogenes* counts were performed on Agar Listeria Ottavani and Agosti (ALOA) (BioMérieux, France) for 24 h to 48 h incubation at 37 °C in aerophilia (ISO 11290-2:2015).

### 2.8. Biogenic Amines (BAs)

BAs were extracted in duplicate with perchloric acid and derivatised with dansyl chloride according to the method described by Alves et al. [25]. The separation of eight biogenic amines, namely, tryptamine, 2-phenylethylamine, putrescine, cadaverine, histamine, tyramine, spermidine and spermine, was performed with a chromatographic reversed-phase column (Thermoscientific RP-18, 5 μm, 250 × 5 µm, Supelco, Bellefonte, PA, USA) with UV detection at 254 nm. Identification of the BAs was performed by comparison of the BAs’ retention times with standard solutions. The quantification of BAs was carried out using 1,7-diaminoheptane as an internal standard, and the amounts of BAs were expressed as milligrams per kilogram.

### 2.9. Colour

The colour analysis was carried out on both fillets, after the texture measurements, using a Chrome Meter CR-400 (Konica Minolta, Osaka, Japan) and the results were recorded as L*, a* and b* coordinates from the CIELab system. L* denotes lightness on a scale of 0 (black) to 100 (white), a* values describe the intensity from green (−) to red (+) and the b* values range from blue (−) to yellow (+). The colorimeter (illuminant condition D_65_ and 2° standard observer) was first calibrated using a calibrating white plate and then four measurements were taken of each fillet (two on the dorsal part and two on the ventral part, i.e., parts 3a and 3b, respectively, presented in Figure 2).

### 2.10. Texture

The texture analysis was carried out using a TA.XTplus analyser (Stable Micro Systems, Surrey, UK); the Texture Profile Analysis (TPA, double compression test) was performed using a 30 kg load cell and an aluminum compression plate of 75 mm diameter (P75). The highest part of both fillets (part 3a in Figure 2) was cut into 20 × 20 mm cubes (one per fillet), which were compressed twice by up to 60% of the original height (12–15 mm) at a constant speed of 1.00 mm/s [14]. The primary characteristics obtained included hardness (maximum force of the compression), springiness (distance of the height detected during the second compression divided by the original compression distance) and cohesiveness (area of work during the second compression divided by the area of work during the first compression). The chewiness was calculated as hardness × cohesiveness × springiness [26].

### 2.11. Sensory Analysis

The sensory assessment was performed in a test room (ISO 8589: 2007) using a quantitative descriptive method [27] and six trained panellists (ages ranged from 30–60 years old; 60% women), selected among the IPMA’s expert panel on fish/seafood, including smoked fish products containing NaCl/KCl combinations. However, these panellists received extra training on salty and bitter taste, as well as on smoked taste/odour and off-flavours in a previous trial carried out with smoked fish salted with 100% NaCl and different NaCl/KCl formulations [15]. Slices of 30 mm wide and 12–15 mm thickness (from part 2 presented in Figure 2) were taken from each fillet, individually wrapped in aluminium foil (food grade), coded and stored at 7 °C until the assessment (within 3 h). The samples were presented to the panellists at room temperature (20 °C) in white dishes and the panel rated the intensity of attributes/descriptors using a 5-point scale (0—absent, 1—slight, 2—moderate (adequate in the case of salty taste), 3—strong, 4—extreme). The samples were assessed in a single session lasting 40–60 min and the scoresheet included clear instructions to ensure adequate rinsing of the mouth and palate (drinking water, eating a small piece of cracker and waiting 5–7 min before tasting another sample). The sensory test was focused on the taste (in particular salty and bitter) but also the smoky odour/taste, off-odours/flavours and texture properties (firmness and succulence) were evaluated.

### 2.12. Statistical Analysis

Statistical analysis was performed using the STATISTICA software version 13 (StatSoft. Inc., Tulsa, OK, USA). The effect of the brine and formulation (NaCl replacement by KCl) on the parameters analysed was tested by factorial analysis of variance (ANOVA) and Tukey’s HSD test. Statistical significance was considered at *p* ≤ 0.05.

## 3. Results and Discussion

### 3.1. Yield

Salting and smoking did not affect the processing yield, which ranged from 78–81% (Table 1). The weight loss of the mackerel (19–22%) was similar to that obtained in other salted and hot-smoked fish species, such as matrinxa (*Brycon cephalus*) (19%) [28].

### 3.2. Proximate Composition

The effect of substitution of NaCl with KCl at different levels on the proximate composition of smoked mackerel is presented in Table 1. Moisture and fat contents did not differ significantly between formulations (*p* ≤ 0.05). The water content found in smoked mackerel formulations dropped by approximately 10% compared with raw fish (70.3 ± 2.9 g/100 g). A similar water content reduction (9%) was reported for smoked trout [29]. The protein content in raw material was 22.7 ± 0.4 g/100 g, while in the smoked products, it was 28–30 g/100 g and, in general, was similar between all formulations. However, significant differences were found in the B formulation (75% NaCl + 25% KCl) (5% brine) vs. A (100% NaCl) (10%), C (50% NaCl + 50% KCl) (10%) and D (25% NaCl + 75% KCl) (5%). Such differences certainly resulted from the variability between individuals since the slight differences in the ionic strength of brines did not justify the impact on the protein solubilisation. Even though the fish were caught in the same area, they had different ages and sizes and, therefore, the content of nutrients, such as protein could be different [30]. Similar contents of protein (26–29 g/100 g) and fat (9–10 g/100 g) were found in commercial smoked mackerel available in, e.g., Polish or American markets [4,31].

### 3.3. Sodium Chloride (NaCl), Sodium (Na), Potassium (K) and Chloride (Cl^−^)

The content of Na, K, NaCl and Cl^−^ is presented in Table 1. Brine and composition significantly affected NaCl, Na and K contents. The Na content found in raw mackerel was 70 mg/100 g, a value similar to those found in the literature for chub mackerel (70–75 mg/100 g) [4,31]. The Na content varied from 0.22 g (0.56 g NaCl) to 0.50 g (1.26 g NaCl) per 100 g in the 5% brine and from 0.31 g (0.77 g NaCl) to 1.04 g (2.60 g NaCl) in the 10% brine. Most of the commercial smoked mackerels available in the Polish and US markets contain 0.8–1.2 g Na (2.0–3.0 g NaCl) [4,31]. According to Norwegian recommendations, which are among the most restrictive globally, the content of salt should be less than 2 g/100 g in hot smoked red and whitefish products [32]. This constraining level was met in six out of eight formulations developed in the study. Thus, only A and B formulations prepared using the 10% brine should be classified as having a high Na (NaCl) content.

Furthermore, as the Na reductions obtained for B, C and D were 10%, 28% and 56% (5% brine), and 22%, 54% and 70% (10% brine), respectively, compared with the controls (A in the 5% and 10% brines), the nutrition claim of *reduced Na/NaCl content* (reduction ≥ 25%) can be applied to four of these products [33].

The content of K in raw fish (0.41 ± 0.01 g/100 g) was comparable to those reported in the literature [4,31]. The NaCl substitution by KCl also resulted in a higher K content, (from 0.50 to 1.37 g/100 g and 0.51 to 2.01 g/100 g in the 5% and 10% brines, respectively, i.e., up to four times more K in Na-reduced products), as expected. Moreover, the Na/K ratio ranged from 0.15 to 0.75 in the products prepared with KCl, i.e., it was in the range recommended by the WHO (<1) for maintaining a healthy cardiovascular condition [34]. Therefore, the consumption of such products can contribute to lower blood pressure in both hypertensive and normotensive people and decrease the stroke risk [35]. The consumption of a usual serving portion (50 g) of smoked mackerel prepared using the 5% and 10% brines would contribute to 7–20% and 7–29%, respectively, of the daily requirement of K for adults (3.5 g) [36]. However, despite the essential role of K in the body, K-containing additives, such as potassium chloride (E508), should be added to food at a “quantum satis” level (i.e., as little as necessary) to avoid excessive K intake [37]. Chloride is another element that influences human health. It contributes to the effect of NaCl on blood pressure. According to the latest European Food Safety Authority (EFSA) recommendations, the dietary reference values (DRVs) for the Cl^−^ range from 1.70 g/day (for children aged 1–3 years) to 3.10 g/day (for adults, including pregnant and lactating women) [38]. The content of Cl^−^ in raw fish was 0.15 ± 0.01 g/100 g, while in the 5% brine, it was 1.4–1.6 g/100 g. A higher amount of Cl^−^ was delivered by smoked mackerel prepared using the 10% brine (2.3–2.7/100 g), which corresponds to 44% of the DRV for adults in one serving (50 g) portion. Hence, the Cl^−^ content was significantly affected by the brine concentration.

### 3.4. pH, Water Activity (a_w_) and Water Holding Capacity (WHC)

The brine and formulation significantly affected a_w_, but had no effect on the pH and WHC (Table 2). The pH value of all samples was 6.1–6.2, which was similar to the pH of raw mackerel in this study (6.2 ± 0.1) and the pH of fresh mackerel (6–7) retrieved from literature data [39]. Moreover, the WHC value for the raw material was 49.1 ± 1.1 g/100 g and ranged from 58–65 g/100 g in smoked formulations. The a_w_ was generally higher in samples prepared with the 5% brine (0.954 ± 0.001) than in those prepared with the 10% brine (0.946 ± 0.004). The a_w_ in raw material was 0.964 ± 0.000. Similar a_w_ values were observed by [15] for smoked salmon (0.963), where no differences for this parameter were noticed by partial (0%, 25% and 75%) replacement of NaCl by KCl.

### 3.5. Total Viable Counts (TVC), Enterobacteriaceae and Listeria Monocytogenes

All formulations in both types of brine showed a highly satisfactory microbiological safety regarding the TVC, *Enterobacteriaceae* and *Listeria monocytogenes* levels. In all smoked mackerel, the counts of *Enterobacteriaceae* and *Listeria monocytogenes* were below the limit of quantification (LOQ) of 10 cfu/g and 100 cfu/g, respectively, performed according to the ISO references. Additionally, all samples achieved the legal requirement for *Listeria monocytogenes* [40]. The TVC in raw mackerel was 2.5 ± 0.4 log cfu/g while after salting and smoking, such counts decreased to 1.2–2.1 log cfu/g (no significant differences between formulations). Similar results were obtained for other hot smoked fish species with reduced salt content, such as salmon [15] or trout [41].

### 3.6. Biogenic Amines (BAs)

All smoked mackerels were considered safe regarding BAs (Table 2). No significant differences were found for cadaverine (3.5–5.3 mg/kg in all formulations). The only difference was noticed for spermidine between A and D (5% brine); however, the *p*-value was close to 0.05. The content of other BAs was below the LOQ: tryptamine (2.70 mg/kg), putrescine (2.02 mg/kg), histamine (1.82 mg/kg), tyramine (2.12 mg.kg) and spermine (1.68 mg/kg). The results obtained for histamine were far from the safety limit criteria of 200 mg of histamine per kg of processed mackerel established in the formal European regulation [42], which are sometimes problematic for commercial smoked mackerel. For example, the level of histamine in smoked fish from different species was above the LOQ for eight out of 36 products, reaching 4300 mg/kg [43]. Similar results were noticed in Poland, where histamine was detected in 15% and 18% of the fresh and smoked fish samples, respectively [44]. The microbial quality of raw material influences the content of the BAs—*Enterobacteriaceae*, the major bacterial group with decarboxylase activity, induces the presence of BAs. *Enterobacteriaceae* and BA profiles indicated the high quality of raw material (below LOQ: tryptamine, putrescine, histamine, tyramine; spermine and cadaverine at 3.98 ± 1.07 mg/kg and 1.73 ± 0.35 mg/kg, respectively) and good hygienic conditions maintained during the processing of the fish.

### 3.7. Colour and Texture

Generally, the colour was similar in all smoked mackerels developed (Table 3). The L* parameter ranged from 49.2 to 55.6 in all formulations and only one significant difference was noticed between the two controls with NaCl only. This probably resulted from the variability between individuals, as comparable variability was observed for commercially available smoked salmon [45]. Additionally, the a* and b* values ranged from 1.6 to 3.3 and 17.0 to 20.0, respectively, and did not differ significantly between smoked mackerel. Therefore, the fish colour was not affected (in general) by the brine and formulation (NaCl replacement by KCl). Similar results were observed for Na-reduced smoked salmon and seabass sausage [14,41].

The hardness, springiness, cohesiveness and chewiness are shown in Table 3. In general, all samples presented similar texture properties, as no significant effects of brine and treatment were observed. Similar results showing no significant differences between the hardness of different NaCl and KCl formulations were obtained for smoked products of other fish species. Fuentes and co-authors noticed no differences in hardness between smoked seabass prepared using 100% NaCl and 50% NaCl + 50% KCl [46]. In a study on smoked trout, the hardness was similar between formulations based on 100% NaCl and 50% NaCl + 50% KCl [47]. In these studies, cohesiveness was also similar between samples of smoked seabass and trout.

### 3.8. Sensory Analysis

**Figure 3:**
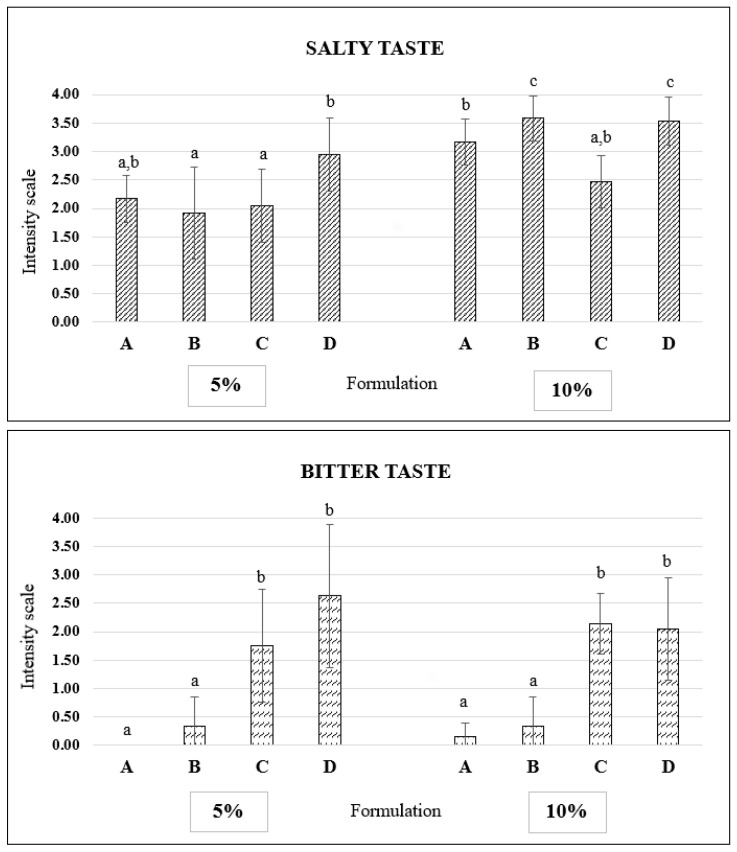
Sensory properties of smoked mackerel formulations. The results are presented as mean values ± SD. Formulations: A—100% NaCl, B—75% NaCl + 25% KCl, C—50% NaCl + 50% KCl and D—25% NaCl + 75% KCl. Different superscript letters indicate significant differences among formulations (*p* < 0.05). Sensory attributes were assessed using a 5-point intensity scale, where 0—absent, 1—slight, 2—moderate (adequate for salty taste), 3—strong and 4—extreme.

Lastly, the results of the sensory analysis allowed for differentiating the formulations developed, as significant differences were noticed for salty taste and bitter taste (Figure 3). The brine concentration and NaCl replacement by KCl significantly affected the salty taste and a slight interaction between these two factors was detected (*p*-value = 0.0439). Thus, the synergistic effect of the KCl on the salty taste enhancement was perceived by the panel, in particular for the products salted in the lower brine concentration (5%). The formulations salted in the 10% brine received higher intensity scores for saltiness (from almost strong/strong to almost extreme intensity) while in the case of the 5% brine, only the formulation containing 75% KCl (D) was scored with a strong salty taste (mean score = 3.0).

The bitter taste was significantly affected only by the higher inclusion of KCl; a bitter taste was not perceived from the A and B formulations in both brines (mean intensity scores in a range of 0.0–0.3), while C and D were scored as moderate bitterness (mean values close to 2) or almost strong (mean score = 2.6). These formulations of both brines did not differ significantly.

Most studies on NaCl reduction clearly identify limited saltiness and the presence of bitterness as a weaknesses for food formulated with KCl [13]. The bitter taste is especially noticeable when the level of substituting NaCl by KCl is higher than 50%. Furthermore, in our study, these parameters were relevant when differentiating the formulations developed. The B formulation in the 5% brine had the most desirable taste attributes, i.e., moderate (i.e., adequate) saltiness and the absence of bitterness. Some authors could reach a higher (50%) replacement of NaCl with KCl when developing, e.g., smoked seabass, salmon or trout [15,47,48], but in other smoked products, the KCl contribution was lower (e.g., 40% in smoked herring) [49]. Moreover, higher KCl addition can be achieved if bitterness-masking ingredients, such as herbs or spices, are integrated into the formulation developed, such as what is performed for seafood products with a full Na content, e.g., [50].

Regarding other sensory properties (data not shown), significant differences in the smoking odour/taste and texture traits were not perceived by the panel. Smoking odour/taste was rated as moderate to strong (mean scores of 2–3) and off-odours/flavours were not perceived. Firmness and succulence were scored as moderate (mean scores of 2) in all formulations. Such results were in line with those found in the instrumental measurement of texture (Table 3).

### 3.9. Market Opportunities and Challenges

The reduction in NaCl content in processed fish products is an important part of the salt reduction strategy in countries with high seafood consumption, such as Iceland, Maldives, Kiribati, Micronesia and Hong Kong, where at least 70 kg of fish and shellfish are consumed every year per person (global average: 20 kg per capita/year) [51,52]. Nonetheless, NaCl reduction in smoked mackerel should not only be the target in these regions, but also for many countries where the product is consumed more often. On the other hand, the price of KCl is approximately 20 times higher than that of NaCl; for example, in Portugal, the prices are approx. 5 vs. 0.25 EUR/kg, respectively (prices valid for consumers in July 2021). Undisputedly, the cost of development of Na-reduced mackerel is higher than the cost of conventional products and even higher when compared with more expensive fish species, such as salmon (where a lower share of salt cost in relation to the total costs of the final product is achieved) [15]. However, taking into account all costs required for the development of smoked fish, the increase in the total cost of Na-reduced mackerel should not be above 5% compared with the product based on NaCl only. Therefore, as nutritionally aware consumers are estimated to represent more than 90% of customers, smoked mackerel with a reduced NaCl content can attract many consumers, particularly those who follow a low-Na diet due to health issues (e.g., with hypertension) [53]. Nonetheless, stability tests and marketing campaigns are still required to ensure a broader utilisation of the product, while the Na reduction strategy employed in this study could be tested with different formulations, including other seafood species.

## 4. Conclusions

Eight formulations with different NaCl and KCl contents were developed and assessed in terms of safety and quality. Most smoked fish presented similar yields, proximate compositions (moisture, fat, protein), textures and colour traits, as well as other quality (WHC, pH) and safety indicators (TVC, *Enterobacteriaceae, Listeria monocytogenes* and biogenic amines). The formulations containing 50 and 75% KCl allowed for obtaining a Na/NaCl reduction in the range of 28–70% compared with those prepared with NaCl only. The most desirable taste attributes, i.e., negligible bitterness and adequate saltiness, were obtained in smoked mackerel prepared with the 5% brine and 75% NaCl + 25% KCl (B formulation), which corresponded to 1.1 g of NaCl, 0.5 g of Na and 0.8 g of K in 100 g of the product. Despite this product’s great potential, it can only be introduced in the market after performing stability tests and marketing campaigns to ensure its broader utilisation. A higher NaCl substitution with KCl could be possible through the addition of (bitterness-masking) ingredients, such as aromas or herbs, that can further help to decrease the Na levels in the final product.

## Figures and Tables

**Table 1:** Yield, moisture, protein, fat, sodium (Na), potassium (K), sodium chloride (NaCl) and chloride (Cl^−^) (g/100 g) in smoked mackerel formulations.

Formulations		Yield	Moisture	Protein	Fat	Na	K	NaCl	Cl^−^
Brine	Formulation	(%)	(g/100 g)	(g/100 g)	(g/100 g)	(g/100 g)	(g/100 g)	(g/100 g)	(g/100 g)
5%	A	80.0 ± 1.20	59.9 ± 1.80	28.4 ± 0.54 ^a,b^	10.4 ± 0.14	0.50 ± 0.02 ^a^	0.50 ± 0.01 ^a^	1.26 ± 0.05 ^a^	1.37 ± 0.03 ^a^
	B	78.0 ± 2.09	61.2 ± 1.90	30.2 ± 1.18 ^b^	9.44 ± 0.75	0.45 ± 0.02 ^a^	0.82 ± 0.02 ^d^	1.14 ± 0.04 ^a^	1.51 ± 0.04 ^a^
	C	79.4 ± 1.33	59.6 ± 1.40	28.4 ± 1.14 ^a,b^	9.16 ± 0.61	0.36 ± 0.02 ^b^	1.10 ± 0.05 ^b^	0.89 ± 0.05 ^b^	1.61 ± 0.16 ^a^
	D	80.7 ± 2.09	59.2 ± 1.98	27.9 ± 0.49 ^a^	9.84 ± 0.11	0.22 ± 0.01 ^c^	1.37 ± 0.04 ^c^	0.56 ± 0.03 ^c^	1.56 ± 0.24 ^a^
10%	A	80.8 ± 0.95	60.1 ± 0.45	27.7 ± 0.26 ^a^	9.32 ± 0.35	1.04 ± 0.05 ^e^	0.51 ± 0.02 ^a^	2.60 ± 0.13 ^e^	2.67 ± 0.11 ^b^
	B	80.8 ± 1.90	59.2 ± 0.26	28.3 ± 0.61 ^a,b^	9.44 ± 0.68	0.81 ± 0.04 ^d^	1.08 ± 0.06 ^b^	2.02 ± 0.11 ^d^	2.74 ± 0.32 ^b^
	C	81.0 ± 1.58	57.5 ± 1.59	27.7 ± 0.46 ^a^	10.3 ± 0.76	0.48 ± 0.01 ^a^	1.44 ± 0.06 ^c^	1.19 ± 0.03 ^a^	2.32 ± 0.13 ^b^
	D	77.7 ± 2.85	58.4 ± 1.46	28.7 ± 0.60 ^a,b^	9.53 ± 0.41	0.31 ± 0.03 ^b^	2.01 ± 0.10 ^e^	0.77 ± 0.06 ^b^	2.49 ± 0.34 ^b^

Results are expressed in wet basis and presented as mean values ± SD. For each column, different superscript letters indicate significant differences between formulations (*p* < 0.05). A—100% NaCl, B—75% NaCl + 25% KCl, C—50% NaCl + 50% KCl and D—25% NaCl + 75% KCl.

**Table 2:** pH, water activity (a_w_), water holding capacity (WHC) (%) and biogenic amines—cadaverine and spermidine (mg/kg).

Formulations		pH	a_w_	WHC	Cadaverine	Spermidine
Brine	Formulation	(-)	(-)	(%)	(mg/kg)	(mg/kg)
5%	A	6.11 ± 0.06	0.953 ± 0.004 ^a,b^	59.0 ± 2.86	4.54 ± 1.41	0.96 ± 0.14 ^a^
	B	6.13 ± 0.11	0.955 ± 0.002 ^a^	61.8 ± 5.25	5.26 ± 1.18	2.43 ± 1.12 ^a,b^
	C	6.15 ± 0.02	0.955 ± 0.001 ^a^	59.2 ± 4.64	4.54 ± 2.26	2.62 ± 0.28 ^a,b^
	D	6.18 ± 0.05	0.954 ± 0.003 ^a,b^	62.6 ± 4.00	4.61 ± 2.38	2.73 ± 0.61 ^b^
10%	A	6.08 ± 0.07	0.940 ± 0.000 ^d^	65.0 ± 3.52	5.12 ± 1.33	1.42 ± 0.53 ^a,b^
	B	6.16 ± 0.04	0.946 ± 0.001 ^c^	61.8 ± 1.04	3.87 ± 0.48	1.01 ± 0.17 ^a^
	C	6.16 ± 0.10	0.949 ± 0.001 ^a,b,c^	57.5 ± 5.54	4.45 ± 1.62	1.24 ± 0.40 ^a,b^
	D	6.19 ± 0.06	0.948 ± 0.004 ^b,c^	61.3 ± 2.43	3.46 ± 2.22	1.26 ± 0.83 ^a,b^

Results are expressed in wet basis and presented as mean values ± SD. For each column, different superscript letters indicate significant differences between formulations (*p* < 0.05). A—100% NaCl, B—75% NaCl + 25% KCl, C—50% NaCl + 50% KCl and D—25% NaCl + 75% KCl.

**Table 3:** Colour and texture properties of the smoked mackerel formulations.

Formulations		Colour			Texture			
Brine	Formulation	L*	a*	b*	Hardness (N)	Springiness (-)	Cohesiveness (-)	Chewiness (N)
5%	A	55.6 ± 1.2 ^b^	2.4 ± 1.0	20.0 ± 2.8	39.8 ± 3.70	0.52 ± 0.02	0.40 ± 0.02	8.40 ± 1.12
	B	52.7 ± 3.0 ^a,b^	3.0 ± 2.2	19.0 ± 1.6	37.0 ± 2.65	0.48 ± 0.01	0.43 ± 0.02	7.89 ± 0.41
	C	52.5 ± 1.8 ^a,b^	2.8 ± 1.9	20.0 ± 2.6	37.4 ± 2.56	0.47 ± 0.02	0.41 ± 0.02	7.20 ± 0.95
	D	54.2 ± 3.6 ^a,b^	3.0 ± 2.5	20.0 ± 4.3	38.3 ± 2.04	0.49 ± 0.06	0.40 ± 0.01	7.55 ± 0.88
10%	A	49.2 ± 5.1 ^a^	3.3 ± 2.3	18.0 ± 3.8	39.2 ± 3.56	0.51 ± 0.05	0.41 ± 0.01	8.57 ± 0.76
	B	51.0 ± 2.6 ^a,b^	2.6 ± 1.5	17.0 ± 2.4	32.7 ± 3.46	0.50 ± 0.04	0.43 ± 0.01	7.37 ± 0.74
	C	52.0 ± 2.7 ^a,b^	2.5 ± 1.5	18.0 ± 3.8	32.8 ± 1.20	0.45 ± 0.06	0.38 ± 0.03	7.91 ± 1.03
	D	53.1 ± 3.8 ^a,b^	1.6 ± 1.5	19.0 ± 4.7	37.2 ± 4.44	0.50 ± 0.02	0.40 ± 0.04	7.51 ± 0.44

Results are presented as mean values ± SD. For each column, different superscript letters indicate significant differences between formulations (*p* < 0.05). A—100% NaCl, B—75% NaCl + 25% KCl, C—50% NaCl + 50% KCl and D—25% NaCl + 75% KCl.

## Data Availability

Data is contained within the article.
